# Intestinal Microbiota: A Novel Target to Improve Anti-Tumor Treatment?

**DOI:** 10.3390/ijms20184584

**Published:** 2019-09-17

**Authors:** Romain Villéger, Amélie Lopès, Guillaume Carrier, Julie Veziant, Elisabeth Billard, Nicolas Barnich, Johan Gagnière, Emilie Vazeille, Mathilde Bonnet

**Affiliations:** 1 Microbes, Intestin, Inflammation et Susceptibilité de l’Hôte (M2iSH) UMR 1071 Inserm/Université Clermont Auvergne, USC-INRA 2018, CRNH Auvergne, F-63000 Clermont-Ferrand, France; amelie.lopes0703@gmail.com (A.L.); guillaume.carrier@icm.unicancer.fr (G.C.); jveziant@chu-clermontferrand.fr (J.V.); elisabeth.billard@uca.fr (E.B.); nicolas.barnich@uca.fr (N.B.); jgagniere@chu-clermontferrand.fr (J.G.); Emilie.VAZEILLE@uca.fr (E.V.); mathilde.bonnet@uca.fr (M.B.); 2 Biologics Research, Sanofi R&D, 94400 Vitry-Sur-Seine, France; 3 Surgical Oncology Department, Institut du Cancer de Montpellier (ICM), Univ Montpellier, 34298 Montpellier, France; 4 Service de Chirurgie Digestive, CHU Clermont-Ferrand, Inserm, Université Clermont Auvergne, 63003 Clermont-Ferrand, France; 5 3iHP, CHU Clermont-Ferrand, Inserm, Université Clermont Auvergne, 63003 Clermont-Ferrand, France; 6 Service d’Hépato-gastro-entérologie, CHU Clermont-Ferrand, Inserm, Université Clermont Auvergne, 63003 Clermont-Ferrand, France

**Keywords:** intestinal microbiota, chemotherapy, probiotics, cancer, radiotherapy, anticancer treatment, surgery, microbiome, adjuvant therapies

## Abstract

Recently, preclinical and clinical studies targeting several types of cancer strongly supported the key role of the gut microbiota in the modulation of host response to anti-tumoral therapies such as chemotherapy, immunotherapy, radiotherapy and even surgery. Intestinal microbiome has been shown to participate in the resistance to a wide range of anticancer treatments by direct interaction with the treatment or by indirectly stimulating host response through immunomodulation. Interestingly, these effects were described on colorectal cancer but also in other types of malignancies. In addition to their role in therapy efficacy, gut microbiota could also impact side effects induced by anticancer treatments. In the first part of this review, we summarized the role of the gut microbiome on the efficacy and side effects of various anticancer treatments and underlying mechanisms. In the second part, we described the new microbiota-targeting strategies, such as probiotics and prebiotics, antibiotics, fecal microbiota transplantation and physical activity, which could be effective adjuvant therapies developed in order to improve anticancer therapeutic efficiency.

## 1. Introduction

Cancer is among the leading causes of mortality worldwide. Although the death rate from cancer in the United-States has declined steadily over the past 25 years, the American Cancer Society has estimated a total of 1,762,450 new cancer cases and 606,880 deaths from cancer to occur in the country in 2019. It is estimated that microorganisms could be associated with 15% to 20% of cancers [1]. In the recent years, evidence from numerous studies has pointed to the role of commensal microbes as key determinants of health or pathologic conditions, including cancer [2]. The human microbiota is made up of trillions of cells, including bacteria, viruses, and fungi. As the largest population of microbes resides in the gut, intestinal microbiota has been widely studied. 

The gut microbiome is involved in many areas of human health, from providing nutrients and vitamins, to protecting against pathogens, immune system development and epithelial mucosa homeostasis [3]. Given its central functions, the role of the gut microbiome as a contributor to colorectal carcinogenesis has been well studied [4,5,6]. However, because of the complex bidirectional relationship between microbiota and host, the pro-carcinogenic mechanisms have only been partially characterized. As summarized by Zitvogel et al. [7], the gut microbiome can contribute to oncogenesis through several mechanisms: a) by a direct oncogenic effect of microorganisms and their products, b) by altering circulating metabolites which, in turn, become pro-carcinogenic, c) by stimulating the synthesis of trophic factors by the host, and finally d) by disrupting host cancer immunosurveillance through the induction of pro-inflammatory and immunosuppressive pathways [7]. In addition to its pro-carcinogenic properties, the intestinal microbiota has been suspected to affect the efficacy of different therapeutic strategies including surgery, chemotherapy, radiotherapy and immunotherapy [8]. Indeed the gut microbiome has been shown to participate in the resistance to a wide range of anticancer treatments [9,10,11]. Interestingly, these effects have been described on colorectal cancer, but also in other types of malignancies developed at a distance from the gut, such as melanoma, ovarian and lung cancers, pancreatic ductal adenocarcinoma, and sarcoma [9,10,11].

In the first part of this review, we summarized the impact of the gut microbiome on the efficacy of various anticancer therapies including chemotherapy, immunotherapy, radiotherapy and surgery. In the second part, we described the microbiota-targeting strategies developed in order to improve anticancer-therapeutics efficiency.

## 2. Intestinal Microbiota Impacts on Efficacy of Cancer Therapies

Recent preclinical and few clinical studies focusing on several types of cancer strongly support the key role of the gut bacteria in the modulation of host response to anti-tumor drugs, especially chemotherapy and immunotherapy [12]. Studies have suggested that gut microbes play a significant role in anticancer therapy response by modulating drug efficacy, abolishing the anticancer effect, and mediating toxicity. Although the mechanisms are not well understood, some of them have been described as the “TIMER” framework mechanism for Translocation, Immunomodulation, Metabolism, Enzymatic degradation and Reduced diversity [12,13]. In this section, we summarized the role of commensal microbes in modulating cancer therapies efficacy, resistance and toxicity.

### 2.1. Chemotherapy

Gut microbiota seem to be implicated on chemotherapy efficacy through numerous mechanisms, including xenometabolism, immune interactions, and altered community structure [13].

**Figure 1:**
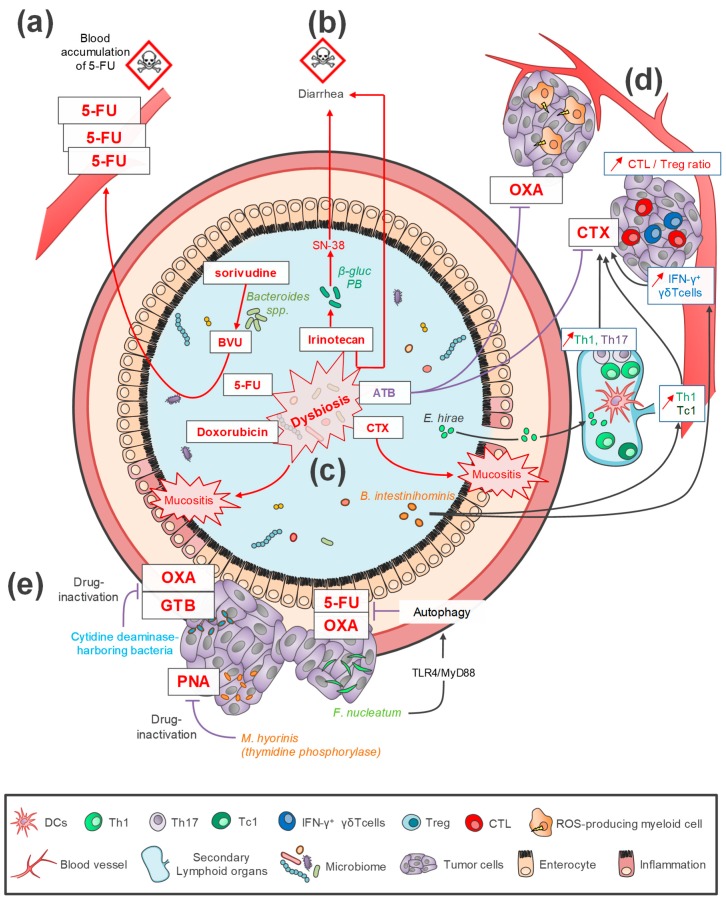
Impacts of intestinal microbiota on chemotherapy toxicity and efficacy. (**a**,**b**) Microbe-mediated xenometabolism could be linked to an increase of chemotherapy toxicity. (**a**) *Bacteroides species* would convert sorivudine into an intermediate component (BVU), which inhibits the degradation of 5-FU, leading to its toxic accumulation in the blood. (**b**) The *β*-glucuronidase-producing bacteria (*β-gluc-PB*) could induce an increase of the irinotecan active metabolite (SN-38) in the gut, which is associated with diarrhea. (**c**) Chemotherapy treatment could induce dysbiosis (microbial composition alteration), as has been noticed for doxorucibin, 5-FU, cyclophosphamide (CTX), and irinotecan. A dysbiosis could impact chemotherapy efficacy and/or side effects (diarrhea, mucositis, etc.). (**d**) Gut microbiota could impact chemotherapy efficacy by immune modulations or/and bacterial translocation to lymphoid organs. OXA chemoresistance has been described in antibiotics (ATB)-treated mice and correlated to a decrease of ROS-producing myeloid anti-tumor cells. The ATB-induced dysbiosis could also decrease the efficacy of CTX. Contrariwise, *Enterococcus hirae*, known to translocate from the gut to lymphoid organs following CTX-induced mucositis, could stimulate the anti-tumor activity of CTX by inducing Th1 and pTh17 responses and increasing the intratumoral cytotoxic T-cells (CTL)/ Tregs ratio. *Barnesiella intestinihominis* could improve systemic amount of Th1 and Tc1 and the intratumoral level of IFN-γ-producing γδ TILs (IFN-δ^+^ γδT cells), leading to an increase of CTX efficacy. (**e**) Intratumoral bacteria could modulate the treatment efficacy. *Mycoplasma hyorhinis* can directly degrade the pyrimidine nucleoside analogues (PNA) through its thymidine phosphorylase activity. Similarly, gemcitabine (GTB) and OXA inactivation could be due to cytidine deaminase-harboring bacteria. The activation of autophagy via the stimulation of the innate immune pathway TLR4/MyD88 by intratumoral bacterial, such as *Fusobacterium nucleatum,* could also be involved in the chemoresistance to 5-FU or OXA.

The gut microbiome is able to directly modify or metabolize some xenobiotics such as anticancer drugs. This microbe-mediated xenometabolism could be linked to an increase of the chemotherapeutic component toxicity, leading to a decrease in treatment efficacy [14,15,16,17]. The most serious potential case of toxicity associated with death was reported in Japan following 5- fluorouracil (5-FU)- sorivudine bi-therapy and involved *Bacteroides* spp. Indeed *Bacteroides* species, dominant members of intestinal microbiota, have a high activity of sorivudine conversion to an intermediate (BVU), which inhibits the degradation of 5-FU and results in its accumulation in the blood and then in a higher toxicity (Figure 1) [15,18]. *Bacteroides species* and other β-glucuronidase-producing bacteria, such as *Faecalibacterium prausnitzii* and *Clostridum* spp., have been associated with the accumulation of irinotecan active metabolite (SN-38) in the gut, leading to diarrhea (Figure 1) [14,16]. Germ Free (GF) mice exhibit less gut damage than conventional mice after irinotecan administration, showing the role of the microbiota on these side effects [19]. Moreover, this could be amplified by the impact of chemotherapeutic drugs on gut and oral microbiota composition itself. Studies in mice have shown that 5-FU therapy induced intestinal dysbiosis with an increase of *Staphylococcus* and *Clostridium* species and a decrease of *Enterobacteriaceae*, *Lactobacillus* and *Bacteroides* [20]. In the same way, severe side effects, such as intestinal mucositis induced by doxorubicin or 5-FU or irinotecan, have been correlated with dysbiosis in the microbiota of the gut and oral cavity [20,21,22]. In conclusion, following treatment, a decrease in microbiota diversity and richness, as well as dysbiosis, could exacerbate severe side effect in murine models of cancer and in cancer patients [23,24,25,26,27,28]. This hypothesis has been reinforced by recent studies showing that microbiome modulation through alimentation or probiotic supplementation could reduce the chemotherapy toxicity and subsequent side effects in mice and humans (see below) [17,29,30,31].

In addition to its role in chemotherapy side effects, gut microbiota also impacts chemotherapy efficacy in pre-clinical models of various subcutaneous solid tumors such as melanoma, lung cancer, colon and sarcoma [10,11,32,33,34]. Two mechanisms have been identified: remote immune modulations or/and bacterial translocation in lymphoid organs. Among the pioneer studies, Iida et al. [10], described the oxaliplatin (OXA) chemoresistance of colon carcinoma and lymphoma in GF or antibiotics-treated mice, in comparison to specific-pathogen-free (SPF) mice. Although the microbial species involved have not been specifically identified, the importance of reactive oxygen species (ROS) producing myeloid anti-tumor cells in the efficacy of OXA has been demonstrated (Figure 1) [10]. Similar results were observed with cyclophosphamide (CTX) treatment, an alkylating agent used for the treatment of lymphomas and solid tumors and known to modulate the immune microenvironment of tumors by reducing regulatory T cells (Tregs) and increasing Th1 and Th17 cells [11,32,33,34]. The CTX efficacy has been shown to be negatively correlated with dysbiosis induced by antibiotic treatment. The authors highlight the positive key role of both *Enterococcus hirae* and *Barnesiella intestinihominis* in the CTX response in non-antibiotic treated mice. *E. hirae* has been shown to translocate from the gut to lymph nodes and to induce Th1 and pathogenic Th17 responses which were mandatory for the anti-tumor activity of CTX (Figure 1)*. B. intestinihominis*, which is accumulated in the colon, improves systemic amount of Th1 and Tc1, polyfunctional CD8^+^ cytotoxic T-cells subpopulation, associated with an increase of IFN-y-producing γδ tumor infiltrating-lymphocytes (TILs) which could also increase the anti-tumor effect of CTX (Figure 1) [11,32,35].

Chemotherapy efficacy can also be directly modulated by intratumoral bacteria through their active metabolic functions (Figure 1) [36,37,38,39,40]. In vitro studies showed a decreased anti-tumoral efficacy of pyrimidine nucleoside analogues in *Mycoplasma hyorhinis*-infected cell lines. Indeed, these anticancer drugs can be directly degraded in tumor cell by mycoplasma thymidine phosphorylase [36]. The cytostatic activity of the drugs was restored by a specific inhibitor of mycoplasma thymidine phosphorylase (Figure 1) [40]. Moreover, it has been shown that cytidine deaminase-harboring bacteria, such as gammaproteobacteria, are involved in the gemcitabine (GTB) and OXA resistance of mice colorectal tumors [39] (Figure 1). The presence of these bacteria and their role on anti-tumoral GTB activity have also been reported in human pancreatic ductal adenocarcinoma [37,38]. Local intratumoral microbiota can also indirectly impact the efficacy of chemotherapy through the modulation of innate immunity. Actually, it has been shown that chemoresistance to 5-FU or OXA was mediated by the activation of autophagy by *Fusobacterium nucleatum* in colorectal cell lines (HCT116 and HT29), xenograft mice models, and colon cancer (CRC) patients [41]. This activation of autophagy was dependent on the stimulation of the TLR4/MyD88 innate immune pathway. A recent study by Zhang et al. confirmed the association between 5-FU chemoresistance and *F. nucleatum* colonization (Figure 1) in CRC patients [42]. This work described the involvement of the TLR4/NF-κB pathway activation which led to the overexpression of BIRC3 (Baculoviral IAP Repeat Containing 3), a member of apoptosis’ inhibitor proteins known to be involved in the chemoresistance of several cancers [42,43].

All these data suggest that the gut microbiota appears to be an essential biomarker to consider for improving chemotherapy regimens. Further clinical studies will be performed to evaluate these innovative markers.

### 2.2. Immunotherapy

**Figure 2:**
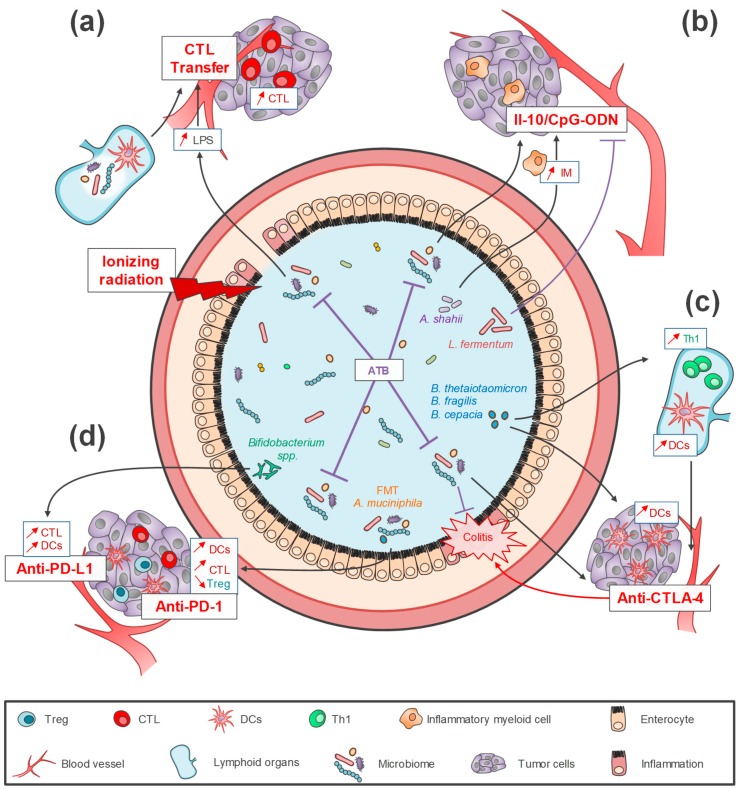
Impacts of intestinal microbiota on efficacy and toxicity of immunotherapy strategies. (**a**) Efficacy of anti-tumor CD8+ T cells (CTL) adoptive transfer (CTL transfer) has been stimulated by gut bacteria translocation into mesenteric lymph node and an increase of systemic LPS concentration induced by the total body irradiation in the murine model. This stimulation has been associated with an increase of CTL recruitment into a tumor microenvironment. (**b**) A decrease of gut bacterial load following antibiotics (ATB) therapy would impair the efficacy of anti-IL-10/CpG oligodeoxynucleotides (ODN) immunotherapy due to the decline of pro-inflammatory cytokine-producing myeloid cells (IM). In the same way, the negative role of *Lactobacillus fermentum* has been shown, meanwhile. *A. shahii* could increase the intratumoral IM and increase ODN efficacy (**c**,**d**) The impact of gut microbiota on ICI efficacy and toxicity via remoted lymphoid and myeloid cells modulation has been noticed. (**c**) *Bacteroides thetaiotaomicron*, *Bacteroides fragilis* and *Burkholderia cepacia* were associated with an increase of Th1 and dendritic cells (DCs) cells in lymphoid organs, leading to an increase in anti-CTLA-4 response. This microbiota-mediated immunotherapy response could be also supported by the recruitment of mature DCs in the tumor microenvironment. In addition, the presence of these specific bacteria could be associated with a decrease of anti-CTLA-4 side effects. (**d**) *Bifidobacterium* spp. was linked to effective anti-PD-L1 response due to the elevation of intratumoral CTL and DCs. The Fecal Microbiota Transplantation (FMT) from responder patients into GF mice could activate similar immune mechanism and improve the response to anti-PD-1 therapy in solid epithelial tumors. (**a**,**b**,**c**,**d**) ATB treatments have been reported to induce a decrease of anti-tumoral efficacy for all of these immunotherapies.

The important role of gut microbiota on immunotherapy efficacy was first shown in two studies which highlighted the involvement of the complex crosstalk between bacteria and immune host response in anti-tumor activity [10,44]. The first study by Paulos et al. [44] showed that the efficacy of anti-tumor CD8^+^ T cells adoptive transfer in a melanoma murine model was strongly increased after the total body irradiation of mice through the translocation of gut bacteria into mesenteric lymph nodes. The microbial lipopolysaccharide (LPS) release induced by irradiation activated innate immune response by TLR4 pathway stimulation and then boosted anti-tumor CD8^+^ T cells, while antibiotic treatment or LPS neutralization (with polymyxin B) were associated with a decrease of anti-tumor response [44]. This effect of gut microbial composition on anti-tumor T cells adoptive transfer has been recently confirmed in B-cell lymphoma, and in cervix and lung tumor mice models [33,45]. The second study, led by Iida et al. [10], described, in MC38 colon carcinoma and B16 melanoma subcutaneous cancer murine models, that antibiotic treatment impaired the efficacy of the anti-IL-10/CpG oligodeoxynucleotides (ODN) immunotherapy. This antibiotic-induced failure of immunotherapy response was due to a decrease of the gut microbiota load, leading to the decline of pro-inflammatory cytokines-producing monocytes in tumor (Figure 2) [10].

More recently, the impact of the gut microbiota on immune checkpoint inhibitor (ICI) therapy efficacy and toxicity has also been largely explored (Figure 2) [46,47,48,49,50,51,52,53]. Although the mechanisms are not well understood, these studies confirm the central role of remote lymphoid and myeloid cells modulation by the gut microbiota [49,51,54,55]. The first studies on ICI focused on sarcoma, melanoma and colon carcinoma murine models [46,47]. One of them showed that GF mice or antibiotic-treated mice were not capable to respond the cytotoxic T-lymphocyte antigen 4 (CTLA-4) inhibitor antibody compared to SPF mice [47]. *Bacteroides thetaiotaomicron*, *Bacteroides fragilis* and *Burkholderia cepacia* were isolated and associated with an effective response to anti-CTLA-4 and with fewer side effects following therapy [47,48]. The restoration of the efficacy after oral feeding of mice with *Bacteroides* spp. was explained by an increase of intratumoral mature DCs with an elevation of Th1 response in tumor-draining lymph nodes. The impact of the microbiome was strongly reinforced by clinical studies which demonstrated that anti-CTLA-4 toxicity can be influenced by gut bacterial composition [48,56]. Another study highlighted that the response to anti-programmed death-ligand 1 (PD-L1) was dependent on the gut microbiota composition and particularly that *Bifidobacterium* spp. was linked to effective response to anti-PD-L1 (Figure 2) [46]. *Bifidobacterium* spp. administration in mice restored the efficacy of this ICI treatment by an increase of IFN-γ^+^CD8^+^T-cells in tumor. Moreover, microbiota composition could predict the status of responder and non-responder patients to anti-programmed cell death 1 (PD-1)/PD-L1 therapy in solid epithelial tumors [49,50,51,52,57]. For example, Gopalakrishnan et al. [49] have shown enrichment of *Faecalibacterium species* in gut microbiome of melanoma patients responding to anti-PD-L1 while *Bacteroides thetaiotaomicron*, *Escherichia coli*, and *Anaerotruncus colihominis* were enriched in non-responders. Fecal Microbiota Transplantation (FMT) from responder patients into GF mice improved ICI efficiency. This effective response seems to be associated with a higher level of intratumoral mature DCs , IFN-γ^+^ CD8^+^ and/or CD4^+^ anti-tumor T cells and a lower amount of intratumoral CD4^+^ FoxP3^+^ Tregs [49,50,51,52]. To demonstrate the role of microbiota on ICI efficacy in melanoma patients, a recent study highlighted that using antibiotics during the first 30 days of ICI therapy was associated with a shorter progression free survival compared to patients who did not receive antibiotherapy [58]. 

These promising results strongly support the inclusion of microbial targeting in anti-tumor immunotherapy strategies to enhance their efficacy, as reported in clinical trials. However, the clinical translation of microbiome effects on immunotherapy is limited by the fact that most preclinical studies have been conducted in mice carrying ectopic syngenic tumor that have already undergone a process of immune editing before transfer into their new host. These models cannot account for the constantly changing interactions between the immune system and tumors during the cancer elimination, equilibrium and escape stages. For a deeper comprehension of the underlying molecular mechanisms, humanized animal models or models of spontaneous tumorigenesis will probably be needed.

### 2.3. Radiotherapy

Radiotherapy is one of common treatments for patients with solid cancers. Ionizing radiation directly induces DNA damage by an energy transfer indirectly through the production of ROS or reactive nitrogen species (RNS) [59,60]. Moreover, radiotherapy can induce local immunogenic effects, such as immunogenic tumor cell death, activating local and systemic inflammation and modulating immunity [61]. Radiotherapy is also responsible of stimulation of the innate immune system. Barker et al. [62] underlined the triggering of a releasing of inflammatory cytokines such as IL-1 and TNF-α and immune cell recruitment after treatment. However, tumor response after radiotherapy remains very heterogeneous, with significant differences from one patient to another and very variable oncologic outcomes. The cause of this heterogeneity remains unclear but recent data have suggested that tumor response could be affected by gut microbiota. The role of intestinal microbiota in radiosensitivity is a new concept generating a lot of interest but with still few original studies leading to convincing results [59]. Emerging preclinical studies performed on mouse models have tried to understand the link between gut microbiota and radioresistance. Cui et al. [63] investigated the effect of circadian rhythm on radiotherapy (with total body exposure) and compared it with the composition of the microbiota. They found that mice with normal 12-h dark/12-h light cycles had a significantly better survival than those with different cycles (8-h dark/16-h light or 16-h light/8-h dark). This was correlated to alterations in gut bacterial communities which could be part of the radioresistance mechanisms. The same team described a correlation between intestinal bacterial communities and radiosensitivity with an antibiotic-treated mouse model. The enteric bacterial composition of treated mice was significantly different from that of control group and the survival rate of antibiotic-treated mice was significantly higher after irradiation [64]. Another hypothesis concerns the link between radioresistance and autophagy regulation, which has already been suggested for nearly 20 years [65]. Digomann et al. [66] found that the expression level of some proteins involved in autophagy was correlated with the clinical prognosis of patients with head and neck squamous cell carcinoma treated with radiochemotherapy [67]. Gut microbiota is also involved in autophagy regulation. Indeed, as described above, the role of *Fusobacterium nucleatum* in chemoresistance through autophagy activation has been shown [41] (Figure 1). To date, no studies on the potential effect of gut microbiota composition on radiosensitivity through autophagy modulation have been published. 

In addition, gut microbiota may affect radio-induced toxicity. Radiotherapy side effects alter quality of life and are an integral part of the treatment decision. For instance in pelvic cancer, in which radiotherapy is a major treatment, Ferreira et al. [68] showed, in a clinical study, a close link between gut microbiota composition and radiation enteropathy. *Clostridium*, *Roseburia* and *Phascolarctobacterium* abundance were significantly increased in patients with radiation enteropathy. Rectal *Roseburia* and *Propionibacterium* were inversely correlated with the rate of IL-15, which was decreased in patients with radiation enteropathy [69]. It is now accepted that ionizing radiation was responsible for microbiota dysbiosis in several pathologies as after pelvic radiation with radiation-induced bowel toxicity [68,69,70,71]. Another clinical study demonstrated a significant alteration of *Firmicutes*/*Bacteroidetes* ratio after pelvic radiation in patients who developed diarrhea [72]. All these studies led to the same conclusion: gut microbial dysbiosis could be a useful biological marker to predict and prevent radiation enteropathy or other complications [68,69,72,73,74]. This hypothesis has been confirmed in a mouse model. Fecal transplantation improved gastro-intestinal tract function in irradiated mice but also protected against radiation-induced death [64]. In addition, it was described in an animal model that neoadjuvant radiation could alter the phenotypic virulence of specific bacteria as *Pseudomonas aeruginosa*, leading to enhanced collagenase activity, junction disruption followed by epithelial cell death and complete monolayer destruction [75].

In conclusion, it is admitted that the intestinal microbiota could be a key player for the modulation of systemic immune response with radiosensitivity and radio-induced toxicity modulation [76]. However, the direct impact of gut microbiota on the efficacy of radiotherapy has not really been demonstrated yet [74]. Further preclinical and clinical studies are needed to identify the microbial populations involved in radioresistance and to make mechanistic assumptions.

### 2.4. Surgery

To our knowledge, the potential impact of gut microbiota on surgery outcomes has only been demonstrated in CRC, probably given the direct interaction between gut microbes and the resection site. The development of preventive strategies to decrease postoperative complications after colorectal surgery remains challenging for physicians treating patients with CRC [77,78,79,80,81]. In particular, anastomotic leaks (AL) are the most common life-threatening postoperative complication. Despite improvements in perioperative medical cares and many studies having been devoted to the technique or the anastomotic configuration, rates of AL have remained largely unchanged over the past decades, reported in 1% to 19% of patients [82,83,84,85]. A potential role of the gut microbiota in the pathogenesis of AL following colorectal surgery is now increasingly obvious [86,87,88,89,90,91]. Blain et al. reported that a parenteral administration of penicillin protected the ischemic bowel from necrosis, a broad spectrum antibiotic being even more protective [92]. The implication of the microbiome in AL was first suggested in the 1950s by Cohn et al., who reported a decrease in AL rates with the intraluminal administration of antibiotics in a dog model of devascularized colonic anastomosis, specifically at the anastomosis site [93]. Since, many studies have investigated the role of an oral antibiotic preparation in elective colorectal surgery and concluded that a potentially significant protective effect of postoperative complications existed, including AL [94]. However, microbial signatures associated with AL and involved mechanisms have been poorly studied. Recently, using 16S MiSeq sequencing on colorectal anastomosis tissue samples, van Praagh et al. reported that AL development was associated with low microbial diversity and correspondingly with a high abundance of the dominant *Bacteroidaceae* and *Lachnospiraceae* families and a low abundance of *Prevotella oralis* [95].

Following colorectal surgery, the restoration of the epithelial barrier integrity is one of the first and main events involved in anastomosis healing [96]. When impaired, underlying bowel tissue layers may be excessively exposed to detrimental intraluminal factors, such as bacteria, which can potentially compromise adequate anastomotic healing, potentially leading to the development of an AL [96]. Studies reported that environmental host signals, such as neoadjuvant therapies or surgery-induced stress, can lead to the activation of virulent bacteria responsible for tissue damages, thus supporting the potential role of gut microbiota alterations in the healing disorders of intestinal anastomoses [97,98]. Recently, Olivas et al. reported a significant increase in AL rates following radiation and low colonic anastomosis in rats inoculated with *Pseudomonas aeruginosa*, a ubiquitous hospital-associated pathogen and colonizer of hospitalized patients [75]. They also found that neoadjuvant radiation could alter the phenotypic virulence of specific bacteria, thus transforming inoculated *Pseudomonas aeruginosa* into a more invasive anastomotic-disrupting phenotype [75]. Moreover, they were able to prevent AL in radiated and inoculated tissue by blocking this transformation using a modified polyethylene glycol. The administration of butyrate enemas postoperatively after left-sided colectomy in a rat model also enhanced anastomotic healing by an increased collagen synthesis and maturation [99,100], potentially due to an inhibition of these pathogenic *Pseudomonas aeruginosa* [101]. In another rat model of AL following low colonic resection, animals which developed AL on devascularized anastomoses were colonized at the anastomotic site by *Enterococcus faecalis* strains with a high collagenase activity, while healed anastomotic tissues were colonized by low collagenase-expressing *Enterococcus faecalis* [102]. These *E. faecalis* strains with a high collagenase activity were able to directly degrade collagen I and indirectly cleave collagen IV by activating host tissue matrix metalloproteinase 9 (MMP9). The inhibition of this collagenase activity, either by the colonic eradication of *E. faecalis* using the intraluminal administration of antibiotics or by the pharmacological suppression of the MMP9 collagenase activity, effectively prevented AL in devascularized anastomoses. Moreover, well vascularized anastomoses inoculated with high collagenase activity *E. faecalis* strains exhibited higher AL rates, similar to that of the devascularized anastomoses.

These findings definitely support the need for further clinical investigations on the host-microbiota interactions following colorectal surgery, and especially during the anastomotic healing process, to identify microbial signatures at risk for postoperative complications and to develop effective gut microbiota-targeted preventive strategies.

## 3. Modulation of the Gut Microbiome to Enhance Therapy Efficacy: from Predictive Potential to Clinical Assays

As described above, intestinal microbiota can provide novel way to enhance the efficacy and to reduce the side effects of current anti-tumoral therapeutic approaches. Several strategies can be considered in order to improve the efficacy of cancer treatment through gut microbiota modulation [9,103,104]. While targeting the gut microbiome has shown some interesting results in the treatment of cancers, the studies demonstrated that improved effects of cancer therapies following microbiota modulation are much more limited [105]. In this second part of the review, we focused on the potential strategies to optimize the microbiota composition in order improve therapy efficiency.

### 3.1. Antibiotics

Antibiotics are effective for treating various infections. Depending on their antimicrobial spectrum and pharmacokinetics, they can also change the composition of the gut microbiota. Antibiotics could be used to eliminate the bacteria that exert a negative effect on cancer therapy efficacy. However, the main issue with antibiotics is the lack of specificity, which in turn, can lead to a dysbiotic microbiota and induce detrimental health effects.

Several studies have assessed the role of an oral antibiotic preparation in elective colorectal surgery and concluded the presence of a potentially significant protective effect from postoperative complications. Rollins et al. compared the impact of the use of oral antibiotics in elective colorectal surgery in a meta-analysis of randomized controlled trials and cohort studies and showed a significant reduction of postoperative complications in patients treated with this therapeutic procedure [94].

Regarding the other types of cancer therapies, published studies have mainly evaluated the impact of antibiotics on ICI efficiency and showed that immunotherapy benefits may be attenuated by the administration of antibiotics [48,51,58,106,107]. A retrospective cohort study by Ahmed et al. [108] recently evaluated whether antibiotic use during anti-PD-1 therapy affected the treatment outcome of patients with cancer (lung, renal, hepatocellular, head and neck, melanoma, urothelial cancers, etc.). Among 60 patients with advanced cancer treated with ICI, 17 patients received antibiotics within 2 weeks before and/or after starting therapy because of diverse microbial infections. It is important to note that there was no statistical difference between the two groups in terms of PD-L1 expression. The study showed a lower response rate in patients who received systemic antibiotics within 2 weeks before or after the first dose of therapy when compared to those who did not. The authors defined the types of antibiotic as follows: narrow spectrum only covers Gram-positive bacteria, while broad-spectrum antibiotics cover Gram-positive and negative bacteria, both aerobic and anaerobic. They reported that patients treated with broad-spectrum antibiotics experienced a lowered response rate in contrast to patients treated with narrow spectrum antibiotics. Overall, patients who did not received broad-spectrum antibiotics enjoyed a longer overall survival compared to those who did [108]. On the contrary, in a retrospective cohort study with 90 patients treated with PD-1 ICI (Nivolumab) for non-small cell lung cancer, Hakozaki et al. [109] reported no significant effect of antibiotic use prior to immunotherapy. However, only 13 patients among the cohort received antibiotics and the results showed a trend toward a negative influence of antibiotic use. The authors suggested that the timing between the treatment with antibiotic and the beginning of Nivolumab therapy might play an important role. Indeed, the microbiota composition can drastically change following the discontinuation of antibiotics [110]. Still, for advanced non-small cell lung cancer, Huemer et al. [106] evaluated the impact of concomitant administration of antibiotics in temporal proximity to initiation of immune-checkpoint inhibitors therapy (one month before or one month after initiation), and their results showed that antibiotics attenuated the benefits of immune-checkpoint inhibitors. However, in their study, Vétizou et al. [47] reported an enhanced effect of CTLA-4 blockade in mice administered with vancomycin, probably because the antibiotic induced the overrepresentation of gram-negative *Bacteroidales* and *Burkholderiales* at the expense of gram-positive bacteria such as *Clostridiales*.

Due to a lack of specificity and the induction of a dysbiosis, more investigations are required to reduce the impact of antibiotic use during cancer treatment. Promising perspectives for probiotic co-administration during antibiotic therapy need to be addressed (see below). As an alternative, highly specific strategies such as phagotherapy could be considered to target some gut bacteria. In addition regarding CRC, a preclinical study has demonstrated the efficacy of pro-carcinogenic bacterial-toxin-targeting using small molecules, suggesting the interest of this innovative concept [111].

### 3.2. Fecal Microbiota Transplantation (FMT)

Fecal transplants come with a lot of unknowns, and given the uncertainties, some scientists argue that testing these approaches in humans is risky. FMT has been widely used in the treatment of resistant *Clostridium difficile* infection with high response rates [112], giving promising perspectives for other pathologies. However, FMT data in the context of cancer therapy are much more limited and data have been mainly obtained on animal models.

Since recent clinical papers suggested a potential involvement of the gut microbiome in influencing the efficacy of both CTLA-4- and PD-1-targeting checkpoint inhibitors [49,50,51], the authors of these publications collected the fecal specimens to characterize the mechanisms involved in resistance of immunotherapy on rodent tumor models. Gopalakrishnan et al. [49] orally transferred the fecal microbiota of responder or non-responder patients to anti-PD-1 therapy into melanoma-bearing GF mice. At day 28 post–tumor inoculation and after treatment, responder patient FMT-treated mice had one-sixth the tumor volume of non-responder patient FMT treated mice. In the same way, Matson et al. [50] transferred the fecal material from three human responders and three non-responders from their study into GF mice, followed by the implantation of melanoma cells. They observed two phenotypes of human-microbiota-colonized mice: one with a fast tumoral growth rate, which included two of the three mouse cohorts reconstituted with fecal material from non-responding patients to anti-PD-1, and one with a slower growing rate, including two of the three mouse cohorts reconstituted with fecal material from responding patients [50].

After showing that antibiotics compromised the therapeutic efficacy of ICI in mice and in cancer patients, Routy et al. [51] recolonized antibiotic-treated mice with fecal microbiota from 4 responding and 4 non-responding patients to therapy. Stool samples from clinical responders conferred sensitivity, whereas those from non-responding patients conveyed resistance to PD-1 blockade. The authors observed that FMT from responding patients but not from non-responding patients into GF mice caused tumor growth delay, accumulation of CXCR3^+^CD4^+^ T cells in the tumor microenvironment, and up-regulation of PD-L1 in splenic T cells after PD-1 blockade. Taken together, these results suggest that manipulating the gut microbiota of patients could influence the outcome of ICI, but trials on patients are necessary to confirm these results.

Several clinical trials incorporating FMT in order to improve cancer therapy are still ongoing [103]. A clinical trial led by Dr. Davar (NCT03341143) is currently assessing the effect of FMT in 20 patients with advanced metastatic melanoma who have undergone treatment with pembrolizumab (anti-PD-1) and have failed or become nonresponsive to the therapy during their treatment. They received a fecal microbiota transplant from patients who had proven to be long-term responders to pembrolizumab, in combination with pembrolizumab treatment. Another clinical trial on 40 melanoma patients who failed immunotherapy by PD-1 blockade started in 2017, led by Dr. Markel (NCT03353402). This study aims to change the gut microbiota of patients with fecal transplants from donors who responded to immunotherapy. However, this trial does not combine FMT with the immunotherapy treatment for which patients were nonresponsive. Other trials using FMT are currently in progress to reduce the adverse effects of anticancer treatments (NCT02928523. Although the clinical relevance of FMT seems promising, leaders in the field are still cautious regarding these trials and are looking for standardized approaches. 

### 3.3. Probiotics and Prebiotics

#### 3.3.1. Probiotics

The concept of probiotic proposed by Metchnikoff and defined by the Food and Agriculture Organization/World Health Organization (FAO/WHO) as “live microorganisms which when administered in adequate amounts confer a health benefit on the host” [113,114], has become an important research field [115]. A recent meta-analysis assessing the efficacy and safety of probiotics in adult and pediatric patients diagnosed with cancer suggests that probiotics may be beneficial, even if the mechanisms underlying antitumor properties remain unclear and further studies are still required [116]. However, although most studies have focused on the effect of probiotics on tumorigenesis inhibition or on cancer therapy-related toxicity [117], only a few studies have assessed the potential of probiotics to enhance the efficiency of cancer treatments [118].

In 1993, a phase III multicentered randomized controlled study on 223 patients with carcinoma of the uterine cervix showed that combining treatment with heat-killed *Lactobacillus casei* strains (LC9018) and radiation therapy enhanced tumor regression through the induction of immune response against cancer cells [119]. Since then, the potential applications of the combination probiotics/cancer treatment have sparked interest for future studies. Indeed, gut microbiota seem to regulate the and the repair of irradiation-induced damages [12]. Ciorba et al. [120] have shown that the probiotic strain *Lactobacillus rhamnosus* GG (LGG) protected the mouse intestinal mucosa against irradiation-related toxicity by driving the repositioning of cyclooxygenase 2-expressing cells. 

In a double-blind placebo-controlled trial including 490 patients, the probiotic preparation VSL#3, which includes eight strains of lactic acid-producing bacteria (*Streptococcus thermophilus, Bifidobacterium breve, Bifidobacterium longum, Bifidobacterium infantis, Lactobacillus acidophilus, Lactobacillus plantarum, Lactobacillus paracasei, Lactobacillus delbrueckii subsp. Bulgaricus*), protected the patients against radiation-induced diarrhea [121]. In line with these studies, a number of ongoing clinical trials currently focus on establishing the role of probiotics administration in preventing or limiting the toxic effects of anticancer therapies [104]. However, most of these studies are focused on reducing the radiation-related side effects, but do not investigate the direct impact of probiotics on therapy efficiency. In a clinical randomized control study, patients treated for a nasopharyngeal carcinoma had an enhanced immune response and a decreased rate of radiation toxicities (oral mucositis) after chemoradiotherapy when they received a combination of probiotics to prevent dysbiosis. Indeed, the probiotic combination increased the number of CD4^+^ T cells, CD8^+^ T cells and CD3^+^ T cells and the incidence of severe grade of oral mucositis was significantly decreased in patients treated with probiotics [122]. It should be noted that recently, a study by Wang et al. [73] showed that administration of the probiotic strain *Lactobacillus reuteri* inhibited the development and progression of ICI therapy-associated colitis in melanoma tumor-bearing mice without affecting the antitumor effect of the immunotherapy.

Regarding chemotherapy, a study by Viaud et al. [11] on tumor-bearing mice focused on the role played by the gut microbiota on CTX treatment. The authors observed a disruption of gut mucosal integrity associated with dysbiosis in CTX-treated animals. While they found an impaired tumor regression in GF mice and in antibiotic-treated mice concomitantly to a reduced CTX-induced Th17 cell conversion, an oral supplementation with *Enterococcus hirae* and *Lactobacillus johnsonii* in antibiotic-treated mice facilitated the restoration CTX-mediated Th17 cell conversion [11]. More recently, the same group showed that oral administration of *Enterococcus hirae* in antibiotic-treated sarcoma-bearing mice restored the CTX anti-tumor efficacy by inducing an improved T cell immune response, as described previously [32]. Altogether, the results of these studies highlight specific commensal strains that could be used as probiotics in combination with CTX therapy to improve treatment efficacy in cancer patients. Concerning immunotherapy, a handful of strains correlating to checkpoint inhibitor efficiency have been tested in GF or SPF animal models of cancer [57]. In 2013, Iida et al. [10] showed that an oral administration of *Alistipes shahii* in mice pre-exposed to antibiotics restored the ability of tumor-associated myeloid cells to produce TNF in animals treated with anti-IL-10R/CpG-ODN therapy. As reported by Sivan et al. [46] in a mouse model of cutaneous melanoma, *Bifidobacterium* were associated with slow tumor growth and beneficial responses to anti-PD-L1 therapy. Oral administration of probiotics containing *Bifidobacterium* to mice harbouring unfavorable gut microbiota increased the anti-tumor efficacy of PD-L1 blockade and nearly abolished tumor growth [46]. In 2015, Vétizou et al. [47] compared the efficacy of anti-CTLA-4 antibody treatment in SPF, antibiotic-treated or GF mice with established ectopic tumors. The treatment had no effect on GF or antibiotic-treated mice when compared to SPF mice. The authors found that the administration of anti-CTLA-4 therapy to SPF mice reduced the relative abundance of *Bacteroidales* and *Burkholderiales*. They also reported that GF and antibiotic-treated mice orally fed *Bacteroides fragilis*, *Bacteroides thetaiotaomicron*, *Burkholderia cepacia* or a combination of *Bacteroides fragilis* and *Burkholderia cepacia* recovered from the anticancer response to CTLA-4 antibodies. Vétizou et al. confirmed the clinical relevance of their findings by identifying patients with microbiome composed by a large proportion of *Bacteroides* species and observed that the prevalence of patients with this microbial composition increased following treatment. As underlined by Bashiardes et al. [9], both studies used the same mouse tumor model but identified different bacterial strains that improved therapeutic response, possibly because they used different ICIs or because of distinct mice microbiota. Finally, Tanoue et al. [52] recently isolated 11 commensal strains from healthy human donor feces that are strong inducers of interferon-γ-producing CD8 T cells in the intestine of GF mice. These strains (*Ruthenibacterium lactatiformans*, *Eubacterium limosum*, *Fusobacterium ulcerans*, *Phascolarctobacterium succinatutens*, *Bacteroides uniformis*, *Bacteroides dorei*, *Paraprevotella xylaniphila*, *Parabacteroides distasonis*, *Parabacteroides johnsonii*, *Parabacteroides gordonii*, and *Alistipes senegalensis*) represent rare low-abundance components of the human microbiome. In MC38 tumor-bearing SPF mice, response to anti-PD-1 or anti-CTLA-4 antibodies was significantly enhanced in animal colonized with the 11 strains. These translational studies suggest that bacteria administration may enhance immune-checkpoint inhibitor therapy and chemotherapy efficacy in several rodent tumor models through the stimulation of dendritic cells to secrete IL-12 and differentiate tumor cytotoxic T lymphocytes [57].

Several clinical trials targeting the gut microbiota with probiotic strains to improve anticancer therapy efficacy are ongoing. In 2012, a randomized, double-blind, placebo-controlled phase III study involving 160 patients in Italy with rectal cancer has been designed to assess the efficacy of the probiotic preparation VSL#3® in increasing the pathological major response rate in patients undergoing concurrent chemotherapy and pelvic radiotherapy, but to our knowledge, no data have been published (NCT01579591). Another ongoing study on 20 breast cancer patients that are given Primal Defense Ultra® Probiotic Formula (*Saccharomyces boulardii*, *Lactobacillus plantarum*, *Bacillus subtilis*, *Bifidobacterium lactis*, *Bifidobacterium bifidum*, *Lactobacillus rhamnosus*, *Bifidobacterium breve*, *Lactobacillus casei*, *Lactobacillus salivarius*, *Lactobacillus acidophilus*, *Lactobacillus brevis*, *Bifidobacterium longum*, and *Lactobacillus paracasei*) 2–4 weeks 3 times a day prior to surgery aims to evaluate the efficacy of presurgical probiotics to influence antitumor immune function (NCT03358511). A phase IV randomized clinical study led by Dr. Correia and completed in 2016 (NCT01609660) showed that daily oral supplementation with *Saccharomyces boulardii* for seven days prior to colorectal resection in CRC patients led to a reduction of inflammatory cytokines in the colonic mucosa without affecting the postoperative infection rates [123]. A clinical trial targeting the gut microbiota with a probiotic to improve immune-checkpoint inhibitor efficiency is currently ongoing. This phase I trial aims to evaluate the effect of the probiotic *Clostridium butyricum* CBM588 strain in combination with Nivolumab and Ipilimumab in treating patients with kidney cancer (NCT03829111). In 2019, a trial aiming to compare the effect of chemotherapy on the survival of patients treated for metastatic CRC and that receive or not a probiotic preparation of Weileshu®, but the trial is not yet recruiting (NCT04021589).

Taken together, all these data give crucial information regarding the potential strains to use in order to improve anticancer-therapy efficacy. However, a deeper understanding of the molecular mechanisms underlying single commensal/probiotic strains effects seems to be the next step to optimize probiotic use in association with cancer treatment. 

#### 3.3.2. Prebiotics and Synbiotics

Many food components act as the actual food for gut bacteria which can metabolize those into tumor suppressive metabolites [124]. The use of prebiotics, known as dietary fibers non-digestible or absorbable by the host and specifically used by gut microbes [125], should increase the colonization and relative expansion of particular bacteria and their specific metabolites, which may have a beneficial effect on anti-tumor treatment. However, the potential effect of prebiotics depends on the presence of beneficial bacteria already in the host gut. Thus, the combination of probiotics and prebiotics, known as synbiotic, seems promising.

As described above, a low diversity of the gut microbiome has been independently associated with poor response to immunotherapy in advanced melanoma patients [49], but also with poor outcomes in patients undergoing allogeneic stem cell transplant [126]. In this context, pre- and synbiotic therapy could be considered prior to and along-side therapy to maintain diversity and improve efficacy [8]. To our knowledge, there is only one published study addressing the question of prebiotic or synbiotic in the improvement of cancer treatment efficacy. Indeed, a Brazilian group evaluated the effect of synbiotics in patients with periampullary cancers undergoing curative or palliative treatment by assessing mortality and postoperative infections (NCT0146877). The data published showed a reduced postoperative mortality and complication rates in patients treated pre- and post-surgery with a mixture including *Lactobacillus acidophilus*, *Lactobacillus rhamnosus*, *Lactobacillus casei* and *Bifidobacterium bifidum* combined with fructooligosaccharides [127]. Moreover, a phase II randomized study started in 2019 aims to investigate the effect of probiotics and prebiotics during the definitive treatment of chemotherapy-radiotherapy for patients with localized anal canal squamous cell cancer to improve treatment effectiveness and clinical outcomes (NCT03870607). Prebiotics or synbiotics might improve oncological outcomes in patients, thus allowing the development of new approaches to increase anticancer therapy efficiency.

### 3.4. Physical Activity

Increasing data also suggest the key role of lifestyle on cancer prognosis, more specifically in CRC patients [128]. Dysbiosis could affect the integrity of skeletal muscle, leading to muscle atrophy [129,130,131,132]. The latter is a key player of morbidity and mortality in various cancer entities [133,134,135,136,137] and an important predictor of overall survival in patients, including CRC [138]. The assumption of a new inter-organ cross-talk, ‘gut microbiota-skeletal muscle’ axis, has recently emerged [129]. Indeed, the restoration of commensal *E. coli* levels in a murine model of intestinal chronic inflammation could prevent muscle atrophy [139]. Moreover, the use of a synbiotic treatment in order to restore a “healthy” microbiota has reduced the proliferation of cancer and muscle cachexia and prolonged the survival of mice [7]. For example, restoration of *lactobacilli* levels counteracted muscle atrophy and decreased systemic inflammation in preclinical models of cancer cachexia [140,141]. These models are characterized by a common microbial signature resulting mainly in an increase in *Enterobacteriaceae* species [142,143], specifically *Klebsiella oxytoca* and in a reduction in three butyrate-producing microbial families (*Ruminococcaceae*, *Lachnospiraceae* and *Porphyromonadaceae*) [144]. Taken together, these preclinical data support that the gut microbiota could be the key player in the intestine-skeletal muscle interconnection and thereby contribute to muscle atrophy in cancer. However, gut microbiota in cancer patients with and without cachexia remains to be explore to reinforce the potential interest of ‘gut microbiota-skeletal muscle’ axis in the care of cancer patients. 

Among complementary therapeutic perspectives, physical activity (PA) emerges as an original and promising approach in cancer context. PA seems to have a significant beneficial impact in terms of diagnosis, recurrence, mortality, therapeutic efficacy, and cancer- and treatment-related adverse effects (cachexia, depression, anxiety, and cognitive problems) [145,146,147,148,149,150]. Specifically for CRC, several observational and experimental studies conducted have recommended exercise as an effective method to prevent CRC and improve the negative effects of cancer and its treatment [151,152]. However, the molecular mechanisms underlying the protective effect of exercise regarding cancer have not yet been defined. The benefit of exercise could be related to its impact on metabolic and/or hormonal dysregulations, adiposity, oxidative stress, inflammation and/or immune impairment, transcriptional misregulation, and mitochondrial dysfunction [128,150,151]. In addition, several studies have indicated that exercise could also modulate the tumor physiologic microenvironment and improve antitumor immunity [153,154]. Taken together, these effects of exercise could have a favorable and lasting impact on tumor growth kinetics and metabolism [155,156]. In addition, other suggested mechanisms could also underly the effects of exercise on tumorigenesis, particularly in the CRC context, such as the modulatory effect of PA on colon transit time [157] and on intestinal microbiota [158]. The impact of PA on gut microbiota has not been widely studied in the literature. For instance, preclinical data showed that several weeks of running induced changes in the microbiota of rodents, depending on exercise modalities [159,160,161,162,163,164]. For example, in preliminary preclinical results of Maillard et al. [159], spontaneous regular PA led to a significant decrease in mesenteric adipose tissue in a murine model, mimicking susceptibility to intestinal inflammation and associated with a change in beta diversity of the microbiota associated with the intestinal mucosa. In elite athletes, the levels of PA were correlated with gut microbial features (composition and functional microbial characteristics), including an increase of α-diversity, short-chain fatty acids production, and microbial metabolic capacity [165,166,167]. The impact of PA on gut microbiota was also suggested by Cronin et al. [168] in sedentary healthy subjects in whom the establishment of a controlled PA led changes in gut microbiota characteristics. However, the impact of PA on gut microbiota in severe pathological contexts such as cancer remains an unexplored topic, especially regarding its potential benefit in reducing the negative effects of both cancer and its treatment. This is one of the goals of the single-blinded randomized controlled trial of Newton et al. [169] designed to measure the impact of a supervised exercise program on gut microbiota and its metabolome in patients with prostate cancer receiving androgen deprivation therapy. 

Considering the potential importance of ‘gut microbiota-skeletal muscle’ axis, the modulation of microbiota by PA could be an innovative idea with significant scientific and socioeconomic impacts. These interactions remain to be explored in the cancer context to reinforce the potential interest of PA in the care of cancer patients.

## 4. Conclusions & Prospects

An increased number of studies have recently highlighted the key role of the gut microbiome to fight cancers by modulating the efficacy of cancer therapies and their side effects. A deep understanding of the complex interactions between host response and the microbiota appears certainly essential, but also provide promising tools to optimize the use of anticancer drugs through the modulation of the microbiome. Although all underlying mechanisms have not been well identified, promising tools will be developed to optimize therapeutic decisions by using intestinal microbiota composition as a prognostic factor in cancers [49,170,171,172,173]. These approaches will, of course, require fast, robust and inexpensive screening methods to assess microbiome composition of patients. Eventually, interventional strategies could be set up to ensure that patients have a favorable gut microbiota and a competent immune system at the onset of and all along, a personalized cancer therapy. In conclusion, intestinal microbiota undoubtedly constitutes a new promising lever for the scientific and clinical communities to improve the efficacy of anticancer therapies not only in digestive tract neoplasia but also in all the other tissues.

## Abbreviations

AL	Anastomotic leaks
ATB	Antibiotic
β-gluc-PB	β-glucuronidase-producing bacteria
BIRC3	Baculoviral IAP Repeat Containing 3
BVU	5-(2-bromovinyl)uracil
CRC	Colorectal cancer
CTLA-4	Cytotoxic T-lymphocyte-associated protein 4
CTX	Cyclophosphamide
DCs	Dentritic cells
FAO/WHO	Food and Agriculture Organization/World Health Organization
FMT	Fecal Microbiota Transplantation
GTB	Gemcitabine
GF	Germ-free
ICI	Immune checkpoint inhibitor
IL	Interleukin
LGG	Lactobacillus rhamnosus GG
LPS	Lipopolysaccharides
MMP	Metalloproteinase
NF-κB	Nuclear factor-kappa B
ODN	oligodeoxynucleotides
OXA	Oxaliplatin
PA	Physical activity
PD-1	Programmed cell death 1
PD-L1	Programmed death-ligand 1
RNS	Reactive nitrogen species
ROS	Reactive oxygen species
SPF	Specific Pathogen Free
Tc	Cytotoxic T cell
Th	T helper cell
TILs	Tumor infiltrating-lymphocytes
TLR	Toll-like-receptor
TNF	Tumor Necrosis Factor
Treg	Regulatory T cell
5-FU	5- fluorouracil
